# Supplementary material for: Paclitaxel drug-coated balloon angioplasty for de novo coronary lesions in an expanded real world clinical setting: the multicenter ALLIANCE registry
Source: Cardiovasc Interv Ther. 2026 Apr 8;41(3):628–38. doi: 10.1007/s12928-026-01280-4 (PMC13279377; doi:10.1007/s12928-026-01280-4)
Supplement: Supplementary file 1 — Supplementary file1 (DOCX 229 KB) [file 12928_2026_1280_MOESM1_ESM.docx]

**Supplementary files**

Table S1. Study Organization

Table S2. Definitions and terminology

Table S3. Study Sites, Principal Investigators, and Number of Enrolled Patients

Table S4. Details of the DCB procedure

Table S5. Antithrombotic regimen

Table S6. Univariate analysis of TLF in various groups

Table S7. Univariate analysis of cd-TLR in various groups

**Table S1. Study organization**

| Principal investigator | Masato Nakamura (Toho University Ohashi Medical Center, Tokyo, Japan). |
| --- | --- |
| Co-PI | Kengo Tanabe (Mitsui Memorial Hospital, Tokyo, Japan) |
| Steering Committee | Kazushige Kadota (Kurashiki Central Hospital, Okayama, Japan), Ken Kozuma (Teikyo University School of Medicine, Tokyo, Japan), Takeshi Muramatsu (Fujita Health University Hospital, Aichi, Japan) |
| Clinical Events Committee | Junya Ako (Kitasato University Hospital, Kanagawa, Japan), Takayuki Ogawa (Jikei University Hospital, Tokyo, Japan),  Jun Yamashita (Tokyo Medical University Hospital) |
| Data Safety Monitoring Board | Takafumi Ueno (Marin Hospital, Fukuoka, Japan),  Kenichi Tsujita (Kumamoto University Hospital, Kumamoto, Japan),  Shinya Sonoda (Japanese Red Cross Karatsu Hospital, Saga, Japan) |
| Study statistician | Yoshitaka Murakami (Toho University School of Medicine). |
| Data management | Shiro Ueda (Medical Edge Inc, Tokyo, Japan). |
| Data monitoring | Akihiko Nishimura (Meditrix Co, Tokyo, Japan). |
| Electronic Data Capture System | Shiro Ueda (Medical Edge Inc, Tokyo, Japan) |

**Table S2. Definitions and terminology**

|  | **Definition** | |
| --- | --- | --- |
| **Target Lesion Failure (TLF)** | - Clinically-driven TLR - Target vessel-related myocardial infarction (Q wave, non Q wave MI) - Cardiac death - Even if it cannot be determined whether myocardial infarction or death is related to the target vessel, it will be considered as target lesion failure in this study protocol. | |
| **Target Vessel Failure (TVF)** | - Clinically-driven TVR - Target vessel-related myocardial infarction (Q wave, non Q wave MI) - Cardiac death - Even if it cannot be determined whether myocardial infarction or death is related to the target vessel, it will be considered as target lesion failure in this study protocol. | |
| **Technical success rate^１）^** | - Technical success was evaluated based on two definitions.  1. Post-procedural residual stenosis of lesion <30% by QCA and absence of dissection beyond NHLBI type C compromising flow. 2. Post-procedural residual stenosis of lesion <40% by QCA without dissection beyond NHLBI type C. | |
| **Clinical procedural success rate** | - Technical success１）with no death or MI noted within 24 hours of the index procedure | |
| **Clinically driven TLR ^2)^** | TLRs that meet the following criteria   - When the stenosis degree of the target lesion is 50% or more on on-site QCA, and ischemic symptoms or functional ischemia are observed (either of the following) - Ischemic symptoms caused by the target lesion are present. - Evidence of ischemia thought to be caused by the target lesion is present during rest or stress testing. - Functional ischemia is present based on invasive diagnostic tests (Doppler flow reserve (FVR), fractional flow reserve (FFR)). - No ischemic symptoms or functional ischemia findings are present, but the target lesion has a stenosis of 70% or more on on-site QCA. | |
| **Periprocedural MI ^3)^** | Perioperative MI is defined as follows, based on SCAI definition ^.^  1) Patients with normal baseline CK-MB values measured within 2 weeks prior to the procedure:  Patients with a peak CK-MB value measured within 48 hours after the procedure that is 10 times or more the ULN of the facility laboratory; or an increase to 5 times or more the ULN accompanied by new pathological Q waves or new persistent LBBB in two or more adjacent leads; or patients without CK-MB measurements but with normal baseline troponin values, and a single troponin (I or T) value measured within 48 hours post-procedure is 70 times or more the ULN of the local laboratory, or an increase to 35 times or more the ULN accompanied by new pathological Q waves in two or more adjacent leads or new persistent LBBB.  2) Patients with elevated baseline CK-MB (or troponin) levels measured within 2 weeks prior to the procedure, and stable or decreasing biomarker levels:  CK-MB (or troponin) levels increased by an absolute increment equal to the recommended values from the most recent pre-procedure values.  3) Patients with elevated CK-MB (or troponin) levels and no evidence of stable or decreased biomarker levels:  CK-MB (or troponin) levels increased by an absolute increase equal to the aforementioned recommended values + new ST elevation or depression + new onset or worsening of heart failure or sustained hypotension, among other clinically relevant signs consistent with MI. | |
| **Frailty ^4,5)^** | Frailty is assessed based on the Japanese version of the Clinical Frailty Scale (CFS version 2.0)  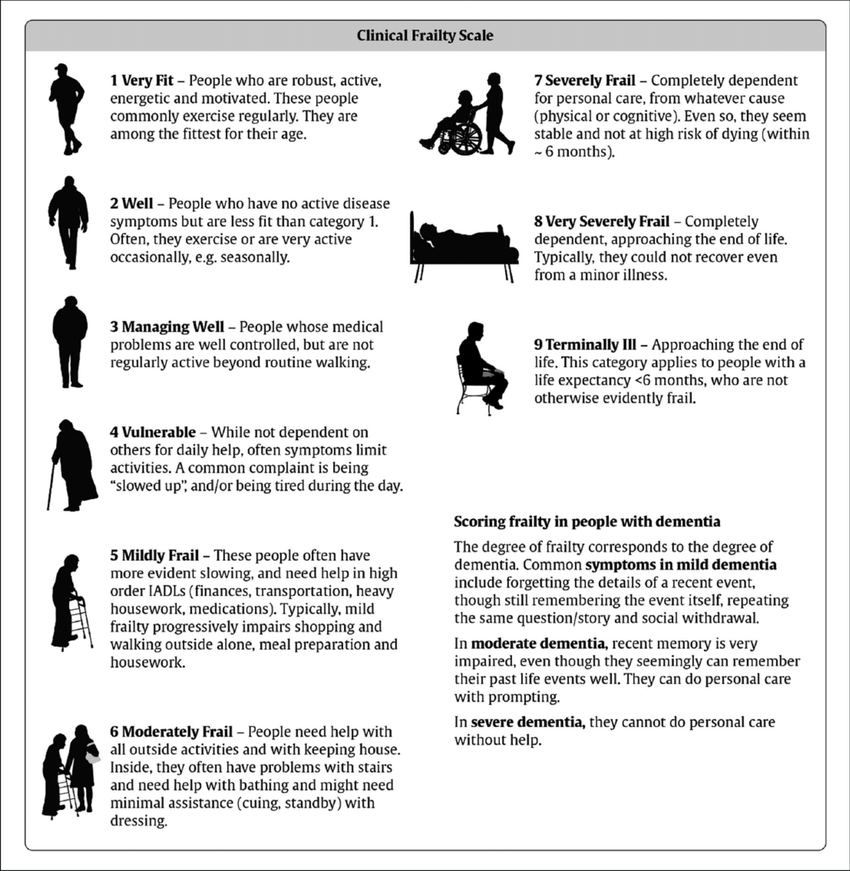 | |
| **Coronary Dissection Grade Based on IVUS Evaluation ^6)^** | Dissection assessed by IVUS is defined according to the following grades based on a substudy of the ADAPT-DES trial  Grade 1: Intimal Dissection  Dissection with a tear limited to the intima  Grade 2: Medical Dissection  Dissection that extends into the media  Grade 3: Intramural Hematoma  Medial dissection but is distinguished by the appearance of blood accumulation within the medial space  Grade 4: Extramedial Injury  Dissection extending beyond the media with blood visible in the perivascular tissue | |
| **Classification of Coronary Dissection by NHLBI ^7)^** | For coronary dissection evaluated by angiography, the following NHLBI definition 2 is used.  Type A: Small radiolucent area within the lumen of the vessel disappearing with passage of the contrast material  Type B: Appearance of contrast medium parallel to the lumen of the vessel disappearing within a few cardiac cycles  Type C: Dissection protruding outside the lumen of the vessel persisting after passage of the contrast material  Type D: Spiral-shaped filling defect with or without delayed run-off of contrast material in the antegrade flow  Type E: Persistent luminal filling defect with delayed run-off of contrast material in the distal lumen  Type F: Filling defect accompanied by total coronary occlusion | |
|  |  |  |

Ref.

1. Fezzi S, Scheller B, Cortese B, Alfonso F, Jeger R, Colombo A, et al. Definitions and standardized endpoints for the use of drug-coated balloon in coronary artery disease: consensus document of the Drug Coated Balloon Academic Research Consortium. Eur Heart J 2025;46: 2498–2519
2. Donald E. Cutlip, Stephan Windecker, Roxana Mehran et al. Clinical End Points in Coronary Stent Trials. A Case for Standardized Definitions。Circulation. 2007; 115:2344-2351.
3. Moussa ID, Klein LW, Shah B et al. Consideration of a new definition of clinically relevant myocardial infarction after coronary revascularization: an expert consensus document from the Society for Cardiovascular Angiography and Interventions (SCAI). J Am Coll Cardiol 2013;62:1563-70.
4. Rockwood K et al. A global clinical measure of fitness and frailty in elderly people. CMAJ 2005:173;489-495.
5. Clinical Frailty Scale © 2005-2020 Rockwood, Version 2.0 (JA). All rights reserved. For permission: [www.geriatricmedicineresearch.ca](http://www.geriatricmedicineresearch.ca). Translated with permission to Japanese by the Japan Geriatrics Society, Tokyo, 2021.
6. N Kobayashi, GS Mintz, B Witzenbichler, et. al. Prevalence, Features, and Prognostic Importance of Edge Dissection After Drug-Eluting Stent Implantation: An ADAPT-DES Intravascular Ultrasound Substudy. Circ Cardiovasc Interv 2016; 9 (7) e003553.
7. Maria D. Radu, Lorenz Räber, Jungho Heo, et al. Natural history of optical coherence tomography-detected non-flow-limiting edge dissections following drug-eluting stent implantation. EuroIntervention 2014; 9:1085-1094.

**Table S3 Study Sites, Principal Investigators, and Number of Enrolled Patients**

| Study site | Principle investigator | No. of patients |
| --- | --- | --- |
| Sapporo Cardiovascular Clinic | Yutaka Tadano | 102 |
| Kokura Memorial Hospital | Kenji Ando | 96 |
| Kyoto Katsura Hospital | Shigeru Nakamura | 82 |
| Japanese Red Cross Musashino Hospital | Takashi Ashikaga | 73 |
| Toyohashi Heart Center | Yoshihisa Kinoshita | 66 |
| Miyazaki Medical Association Hospital | Nehiro Kuriyama | 66 |
| Hiratsuka Kyosai Hospital | Yuko Ohnishi | 66 |
| Bell Land General Hospital | Toru Kataoka | 61 |
| Kurashiki Central Hospital | Kazushige Kadota | 56 |
| Chikamori Hospital | Koji Nishida | 55 |
| Toho University, Ohashi Medical Center | Raisuke Iijima | 49 |
| Saiseikai Fukuoka General Hospital | Masatsugu Nozoe | 49 |
| Japanese Red Cross Matsuyama Hospital | Kunio Morishige | 49 |
| Japanese Red Cross Tokushima Hospital | Takefumi Takahashi | 46 |
| Showa Medical University Fujigaoka Hospital | Hiroshi Suzuki | 41 |
| Kansai Rosai Hospital | Takayuki Ishihara | 40 |
| Tenjinkai Shin-Koga Hospital | Tomohiro Kawasaki | 35 |
| Sapporo-Kosei General Hospital | Yasumi Igarashi | 34 |
| Sakurabashi Watanabe Advanced Healthcare Hospital | Atsunori Okamura | 34 |
| Sakakibara Heart Institute | Mamoru Nanasato | 34 |
| Osaka Rosai Hospital | Masami Nishino | 34 |
| NHO Kyushu Medical Center | Yoshinobu Murasato | 33 |
| Gifu Heart Center | Hitoshi Matsuo | 33 |
| Hoshi General Hospital | Ryoji Koshida | 32 |
| The Cardiovascular Institute | Shunsuke Matsuno | 31 |
| Tokyo Metropolitan Tama Medical Center | Toshiaki Isogai | 30 |
| Sapporo Higashi Tokushukai Hospital | Seiji Yamazaki | 30 |
| Matsunami General Hospital | Hiroki Kondo | 28 |
| Fujita Health University Hospital | Takeshi Muramatsu | 27 |
| NTT Medical Center Tokyo | Jiro Ando | 26 |
| Juntendo University Shizuoka Hospital | Satoru Suwa | 25 |
| Iwatsuki Minami Hospital | Yasuyuki Maruyama | 23 |
| Iwate Medical University Hospital | Masaru Ishida | 22 |
| Fukuoka Sanno Hospital | Hiroyoshi Yokoi | 21 |
| Mitsui Memorial Hospital | Kengo Tanabe | 20 |
| Yokohama City University Medical Center | Kiyoshi Hibi | 19 |
| Ageo Central General Hospital | Nobuhiko Ogata | 18 |
| Shonan Kamakura General Hospital | Yutaka Tanaka | 18 |
| Sendai Kousei Hospital | Shintaro Honda | 17 |
| St. Marianna University Hospital | Yasuhiro Tanabe | 17 |
| Niigata City General Hospital | Kazuyoshi Takahashi | 17 |
| St. Luke’s International Hospital | Taku Asano | 16 |
| Tokyo Women’s Medical University Hospital | Junichi Yamaguchi | 15 |
| Gunma University Hospital | Hideki Ishii | 14 |
| Aichi Medical University Hospital | Tetsuya Amano | 13 |
| Tokyo Rosai Hospital | Norihiko Kougame | 12 |
| Tokyo Bay Urayasu Ichikawa Medical Center | Kotaro Obunai | 11 |
| Ogaki Municipal Hospital | Itsuro Morishima | 10 |
| Jichi Medical University Saitama Medical Center | Kenichi Sakakura | 10 |
| Kindai University Hospital | Gaku Nakazawa | 9 |
| Tosei General Hospital | Hiroshi Asano | 9 |
| Teikyo University Hospital | Ken Kozuma | 6 |
| Suzuka General Hospital | Satoshi Ota | 4 |
| Osaka Saiseikai Nakatsu Hospital | Yoichi Kijima | 4 |
| Tokai University Hospital | Yuji Ikari | 4 |
| Gunma Prefectural Cardiovascular Center | Ren Kawaguchi | 3 |

Table S4. Details of the DCB procedure

| Lesion preparation (N=1984) | |
| --- | --- |
| Present | 1972/1984 (99.4) |
| None | 12/1984 (0.6) |
| Angiographic coronary dissection after lesion preparation (on site evaluation) | |
| None | 1360/1972(69.0) |
| Present | 612/1972(31.0) |
| NHLBI type A | 273/612(44.6) |
| NHLBI type B | 272/612(44.4) |
| NHLBI type C | 54/612(8.8) |
| NHLBI type D | 9/612(1.5) |
| NHLBI type E | 3/612(0.5) |
| NHLBI type F | 1/612(0.2) |
| Angiographic coronary dissection after DCB (on site evaluation) | |
| None | 1717/1984(86.5) |
| Present | 267/1984(13.5) |
| NHLBI type A | 108/267(40.4) |
| NHLBI type B | 107/267(40.1) |
| NHLBI type C | 32/267(12.0) |
| NHLBI type D | 10/267(3.7) |
| NHLBI type E | 6/267(2.2) |
| NHLBI type F | 4/267(1.5) |
| DCB | |
| DCB Balloon size (min, median, max) | 2.65±0.56 (2.0, 2.5, 4.0) |
| No. of utilized DCB balloons | |
| 1 | 1844/1984(92.9) |
| 2 | 129/1984 (6.5) |
| 3 | 7/1984 (0.4) |
| 4 | 4/1984 (0.2) |
| Bail-out use of DES | 22/1984 (1.1) |
| 1 DES | 21 |
| 2 DES | 1 |
| On site QCA analysis (n=1875) | |

| Pre Ref. | 2.50±0.72 |
| --- | --- |
| Pre % DS | 75.17±15.58 |
| Pre MLD | 0.78±0.46 |
| Lesion length | 16.66±11.06 |
| Post Ref. | 2.59±0.70 |
| Post % DS | 26.13±15.13 |
| Post MLD | 1.94±0.66 |

NHLBI: National Heart, Lung, and Blood Institute, DES: drug eluting stent, DCB: drug coated balloon, Ref: reference, DS; diameter stenosis, DS: diameter stenosis, MLD: minimum lumen diameter

Supple. Table 5. Antithrombotic regimen

|  |  | At discharge  n=1794 | 6 months n=1665 | 12 months n=1661 |
| --- | --- | --- | --- | --- |
| Antiplatelet drug | Aspirin | 1604 (89.4) | 922 (55.4) | 702 (42.3) |
|  | Clopidogrel | 481 (26.8) | 386 (23.2) | 357 (21.5) |
|  | Prasugrel | 1260 (70.2) | 912 (54.8) | 839 (50.5) |
|  | Ticlopidine | 4 (0.2) | 4 (0.2) | 4 (0.2) |
| DAPT | | 1559 (86.9) | 654 (39.3) | 396 (23.8) |
| Anticoagulant | Warfarin | 24 (1.3) | 30 (1.8) | 30 (1.8) |
|  | DOAC | 191 (10.6) | 187 (11.2) | 178 (10.7) |

DAPT, dual antiplatelet treatment; DOAC, direct oral anticoagulant.

Supple Table 6. TLF in various groups and univariate analysis

|  |  |  | Univariate | | |
| --- | --- | --- | --- | --- | --- |
|  |  | Event | HR | 95% CI | P-value |
| Age | ≥64 | 19/515 (3.7%) | 1.13  1.66 | 0.61 - 2.09  0.97 - 2.83 | 0.709  0.065 |
|  | 65-74 | 21/515 (4.1%) |  |  |  |
|  | ≥75 | 45/764 (5.9%) |  |  |  |
| Sex | Male | 63/1369 (4.6%) | 1.14 | 0.70 - 1.86 | 0.591 |
|  | Female | 22/425 (5.2%) |  |  |  |
| Diabetes mellitus | No | 34995 (3.4) | 1.88 | 1.22 - 2.90 | 0.004 |
|  | Yes | 51/799 (6.4%) |  |  |  |
| Hyperlipidemia | No | 27/399 (6.8%) | 0.58 | 0.37 - 0.92 | 0.021 |
|  | Yes | 58/1395 (4.2%) |  |  |  |
| Heart failure | No | 68/1575 (4.3%) | 1.88 | 1.11 - 3.20 | 0.020 |
|  | Yes | 17/219 (7.8%) |  |  |  |
| Hemodialysis | No | 68/1646 (4.1%) | 2.94 | 1.73 - 5.01 | <.001 |
|  | Yes | 17/148 (11.5%) |  |  |  |
| Previous PCI | No | 47/927 (5.1%) | 0.86 | 0.56 - 1.32 | 0.499 |
|  | Yes | 38/867 (4.4%) |  |  |  |
| Previous MI | No | 65/1350 (4.8%) | 0.94 | 0.57 - 1.55 | 0.805 |
|  | Yes | 20/444(4.5%) |  |  |  |
| Acute coronary syndrome | No | 49/1281 (3.8%) | 1.91 | 1.25 - 2.94 | 0.003 |
|  | Yes | 36/513 (7.0%) |  |  |  |
| Bifurcated lesion | No | 651308 (5.0%) | 0.82 | 0.50 - 1.35 | 0.430 |
|  | Yes | 20/488 (4.1%) |  |  |  |
| Ostial lesion | No | 51/1393 (3.7%) | 2.35 | 1.52 - 3.62 | <0.001 |
|  | Yes | 34/401 (8.5%) |  |  |  |
| Calcified lesion | No | 46/1233 (3.7%) | 1.95 | 1.27 - 2.99 | 0.002 |
|  | Yes | 39/561 (7.0%) |  |  |  |
| Diffuse lesion | No | 60/1317 (4.6%) | 1.18 | 0.74 - 1.88 | 0.489 |
|  | Yes | 25/477 (5.2%) |  |  |  |
| DCB balloon size | <3.0㎜ | 45/1120 (4.0％) | 1.50 | 0.98-2.29 | 0.064 |
|  | ≥3.0㎜ | 40/674 (5.9%) |  |  |  |
| DCB dilatation pressure | <6 atm | 6/122 (4.9%) | 0.97 | 0.42 - 2.23 | 0.947 |
|  | ≥6 atm | 79/1672 (4.7%) |  |  |  |
| DCB dilatation time | <60 sec | 31/555 (5.6%) | 0.76 | 0.49 - 1.18 | 0.218 |
|  | ≥60 sec | 54/1239 (4.4%) |  |  |  |
| Coronary dissection after DCB | No | 73/1547 (4.7%) | 1.04 | 0.56 - 1.91 | 0.902 |
|  | Yes | 12/247 (4.9%) |  |  |  |

PCI; percutaneous coronary intervention, MI: myocardial infarction, DCB: drug coated balloon.

Supple Table7. Cd-TLR in various subgroups and univariate analysis

|  |  |  | Univariate analysis | | |
| --- | --- | --- | --- | --- | --- |
|  |  | Event | HR | 95% CI | P-value |
| Age | ≧64 | 14/515 (2.7%) |  |  |  |
|  | 65-74 | 16/515 (3.1%) | 1.17 | 0.57 - 2.39 | 0.677 |
|  | ≥75 | 22/764 (2.9%) | 1.11 | 0.57 - 2.16 | 0.769 |
| Sex | Male | 42/1369 (3.1%) | 0.78 | 0.39- 1.56 | 0.483 |
|  | Female | 10/425 (2.4%) |  |  |  |
| Diabetes mellitus | No | 19/995 (1.9%) | 2.18 | 1.24 - 3.83 | 0.007 |
|  | Yes | 33/799 (4.1%) |  |  |  |
| Hyperlipidemia | No | 13/399 (3.3%) | 0.81 | 0.43 - 1.52 | 0.513 |
|  | Yes | 39/1395 (2.8%) |  |  |  |
| Heart failure | No | 46/1575 (2.9%) | 0.99 | 0.42 - 2.31 | 0.975 |
|  | Yes | 6/219 (2.7%) |  |  |  |
| Hemodialysis | No | 40/1646 (2.4%) | 3.57 | 1.87 - 6.81 | <0.001 |
|  | Yes | 12/148 (8.1%) |  |  |  |
| Previous PCI | No | 26/927 (2.8%) | 1.07 | 0.62 - 1.84 | 0.814 |
|  | Yes | 26/867 (3.0%) |  |  |  |
| Previous MI | No | 42/1350 (3.1%) | 0.73 | 0.36 - 1.45 | 0.361 |
|  | Yes | 10/444 (2.3%) |  |  |  |
| Acute coronary syndrome | No | 34/1281 (2.7%) | 1.38 | 0.78 - 2.44 | 0.270 |
|  | Yes | 18/513 (3.5%) |  |  |  |
| Bifurcated lesion | No | 37/1306 (2.8%) | 1.08 | 0.59 - 1.97 | 0.802 |
|  | Yes | 15/488 (3.1%) |  |  |  |
| Ostial lesion | No | 27/1393 (1.9%) | 3.28 | 1.90 - 5.65 | <.001 |
|  | Yes | 25/401 (6.2%) |  |  |  |
| Calcified lesion | No | 26/1233 (2.1%) | 2.32 | 1.35 - 4.00 | 0.002 |
|  | Yes | 26/561 (4.6%) |  |  |  |
| Diffuse lesion | No | 37/1317 (2.8%) | 1.14 | 0.63 - 2.08 | 0.663 |
|  | Yes | 15/477 (3.1%) |  |  |  |
| DCB balloon size | ＜3.0mm | 25/1120（2.2％） | 1.82 | 1.06 – 3.13 | 0.031 |
|  | ≥3.0mm | 27/674（4.0％） |  |  |  |
| DCB dilatation pressure | <6 atm | 4/122 (3.3%) | 0.87 | 0.32 - 2.46 | 0.816 |
|  | ≥6 atm | 48/1672 (2.9%) |  |  |  |
| DCB dilatation time | <60 sec | 18/555 (3.2%) | 0.82 | 0.46 - 1.45 | 0.487 |
|  | ≥60 sec | 34/1239 (2.7%) |  |  |  |
| Coronary dissection after DCB | No | 43/1547 (2.8%) | 1.33 | 0.65 - 2.73 | 0.435 |
|  | Yes | 9/247 (3.6%) |  |  |  |

PCI; percutaneous coronary intervention, MI: myocardial infarction, DCB: drug coated balloon.

Supple Table 8: Comparison with historical large-scale registries

| Name of trial | Sequent please World Wide Registry | EASTBOURNE  Registry | ALLIANCE  registry |
| --- | --- | --- | --- |
| Trial design | Prospective multicenter | Prospective multicenter | Prospective multicenter |
| No of sites enrolled | 75 | 38 | 57 |
| Enrolled period | Feb.2008～  Nov.2011 | Sep. 2016～  Nov. 2020 | July 2023～  Feb. 2024 |
| DCB | Paclitaxel DCB | Sirolimus DCB | Paclitaxel DCB |
| Study device | Sequent please | MagicTouch | Sequent please NEO/Agent |
| Number of Patients | 2,095 | 2,123 | 1794 |
| ISR/coronary artery disease | 1523/572(72.7%/27.3%) | 910/1173(44%/56%) | 0/1794(0%/100%) |
| Number of treated lesions | 2,234 | 2,440 | 1984 |
| CAD lesions | 559 | 1284 | 1984 |
| ACS | 82(16.7%) | 540(46.0%) | 513(28.6%) |
| Non-small vessel | Limited to small vessel | Limited to small vessel | ≥3mm DCB balloon 674(34.0%) |
| Imaging guidance | N/A | N/A | 1755(88.5%) |
| Bail out stenting | DCB+BMS 106(23.4%) | 112 (8.7%) | 22(1.1%) |
| TLF or MACE | MACE* at 9 months 2.6%* | MACE* at 1 year 4.9% | TLF at 1 year 4.7% |
| TLR | DCB alone 1.0%*/  DCB+BMS 2.4%* | TLR 2.0% | cd-TLR 2.9% |
| Enrolled country | Europa | Europa and Asia | Japan |

* MACE: cardiac death, MI, and TLR

DCB: drug coated balloon, ISR: in-stent restenosis, ACS: acute coronary syndrome, N/A: not available, TLF: target lesion revascularization, MACE: major adverse cardiac event, TLR: target lesion revascularization, cd-TLR: clinically driven target lesion revascularization, BMS: bare metal stent.
